# PTK7: an underestimated contributor to human cancer

**DOI:** 10.3389/fonc.2024.1448695

**Published:** 2024-10-15

**Authors:** Zhipeng Jin, Tianyu Guo, Xue Zhang, Xin Wang, Yefu Liu

**Affiliations:** ^1^ Department of Hepatopancreatobiliary Surgery, Liaoning Cancer Hospital & Institute, Shenyang, Liaoning, China; ^2^ Central Laboratory, Liaoning Cancer Hospital & Institute, Shenyang, Liaoning, China

**Keywords:** PTK7, cancer, biomarker, prognosis, anti-cancer therapy, diagnosis

## Abstract

Protein tyrosine kinase 7 (PTK7) is an evolutionarily conserved transmembrane receptor and a specialized tyrosine kinase protein lacking kinase activity. PTK7 has been found to be strongly associated with a variety of diseases, including cancer. In this review, we will provide a comprehensive overview of the involvement of PTK7 in human cancer, focusing on the changing research landscape of PTK7 in cancer research, the molecular mechanisms of PTK7 involved in cancer progression, the targetability of PTK7 in cancer therapy, and the potential application of PTK7 in cancer management, thus demonstrating that PTK7 may be an underestimated contributor to human cancer.

## 1 Introduction

**Figure 1:**
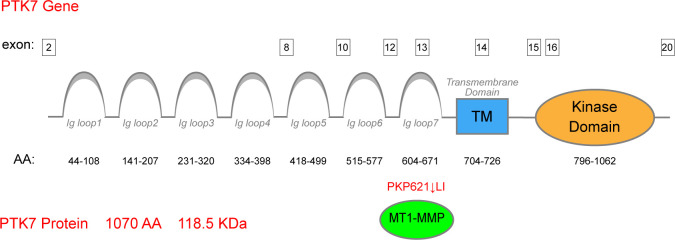
Structure of the PTK7 protein. PTK7 comprises an extracellular domain with seven Ig-like loops, membrane-penetrating structural domains, and homologous structural domains of the RTK family lacking catalytic activity. MT1-MMP serves as the principal sheddase of PTK7, directly cleaving the exposed PKP(621)↓LI sequence of the seventh Ig-like domain.

Protein tyrosine kinase 7 (PTK7) is an evolutionarily conserved transmembrane receptor that belongs to the receptor tyrosine kinase (RTK) family and is a specialized tyrosine kinase protein that lacks kinase activity (1, 2). PTK7 was initially identified as a gene upregulated in colon carcinoma cells and was, therefore, named colon carcinoma kinase 4 (CCK-4) (3). A previous study has clarified that the PTK7 gene is located on the short arm of human chromosome 6 (6p21.1) (4). As for the genomic structure of PTK7, it has been documented that PTK7 is organized into 20 exons (5). The PTK7 protein consists of an extracellular domain with seven immunoglobulin (Ig)-like loops, membrane-penetrating structural domains, and homologous structural domains of the RTK family that lack catalytic activity. MT1-MMP is a principal sheddase of PTK7, which directly cleaves the exposed PKP(621)↓LI sequence and generates an N-terminal, soluble PTK7 fragment (sPTK7) (6–10) (**Figure 1**). As for the location of PTK7, it is currently believed that it is mainly localized at the plasma membrane and has its extracellular domain exposed at the cell surface (11).

In the 1990s, PTK7 expression was found to be associated with the formation of neural crest and inner ear in *Drosophila* and mouse models (12, 13). Subsequent research has revealed its impact on various facets of cell-cell communication and movement, and its crucial roles in the embryonic development of *Drosophila* and even vertebrates have been re-emphasized (14). In the 21st century, PTK7 has been increasingly recognized for its role in controlling tissue morphogenesis and patterns through its effects on cell polarity, migration, tissue regeneration, and wound healing (12, 13, 15–17). These discoveries have highlighted its pivotal role in development, which has also been observed in humans. For instance, mutations in PTK7 have been linked to conditions such as scoliosis and neural tube closure defects (18–20). The multitude of publications on PTK7’s biological functions over the past two decades suggest that its functions are diverse, with the aforementioned discoveries being just the beginning.

Recent research has shed light on the main mechanisms through which PTK7 exerts its biological functions. It is now widely acknowledged that Wnt signaling serves as an important mediator for PTK7 in regulating organism functions (21–25). PTK7 can interact with various Wnt receptors, thereby influencing both canonical and non-canonical Wnt signaling. Notably, its involvement in the planar cell polarity (PCP) pathway, which determines cell orientation in the epithelial cell plane, showcases its contribution to non-canonical Wnt signaling. While the exact role of PTK7 in canonical Wnt signaling remains debatable, existing evidence indicates that PTK7 plays pivotal roles at the intersection of Wnt signaling as a co-receptor involved in determining Wnt signaling outcomes (14, 25). Furthermore, PTK7 serves as a molecular switch between signaling pathways beyond Wnt, as demonstrated by its involvement in VEGF signaling (14). Research has shown that PTK7 forms a receptor complex with Flt-1 (VEGFR1) and plays a crucial role in Flt-1-mediated angiogenesis (16). Additionally, PTK7 expression in perivascular monocytes can induce VEGFR2 and ANG1 expression, contributing to vascular stabilization during angiogenesis (26). Meanwhile, PTK7 can regulate the activity of KDR biphasically (27). However, the role of PTK7 in regulating angiogenesis extends beyond this, with many studies indicating that PTK7 exerts its biological functions in various other ways not limited to the mechanisms mentioned above, indicating the inadequacy of a few words to summarize the role of PTK7 in organisms comprehensively.

Cancer, being one of the most lethal diseases, remains a primary focus of both basic and clinical research. The intricate molecular mechanisms underlying cancer development necessitate the identification of effective therapeutic targets in order to improve clinical outcomes for cancer patients. Given the significant roles of Wnt signaling and VEGF signaling in cancer (28–35), there is an increasing interest in exploring the role of PTK7 in cancer (36). Indeed, studies have shown that PTK7 plays crucial roles in various cancer types, such as lung cancer (37), breast cancer (38, 39), esophageal cancer (40), and colorectal cancer (41, 42). Despite the absence of globally approved anticancer drugs targeting PTK7, various drug types in developmental stages, including antibody-drug conjugates (ADCs), chimeric antigen receptor T-cell immunotherapy (CAR-T), and nanoantibodies, highlight the clinical translational potential of therapeutic strategies centered on targeting PTK7 (43–46).

In this review, we first present the current status of PTK7 research in human cancers to demonstrate the prevalence of PTK7 in various cancer types and its research progress. Subsequently, we will focus on the role of PTK7 in different cancer types, including its expression patterns, influence on cancer cell behavior, and potential regulatory mechanisms. Finally, we will explore the performance of PTK7 in cancer diagnosis, treatment, and prognostic assessment to evaluate its value in the clinical management of cancer.

## 2 The heat of research on PTK7 in human cancer

**Figure 2:**
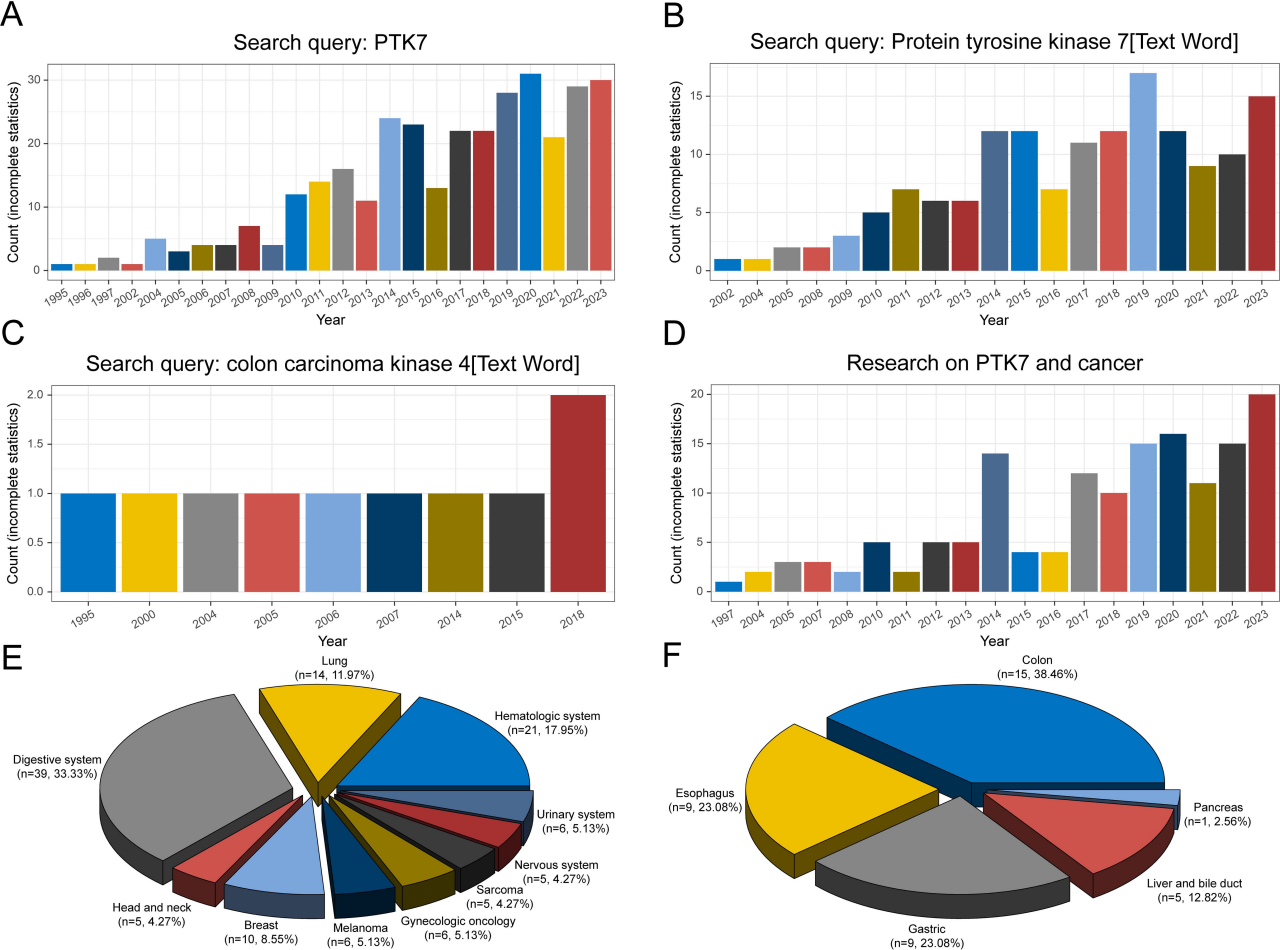
Counts of studies on PTK7 retrieved in PubMed under different search conditions. **(A)** Search keyword “PTK7”; **(B, C)** Search keyword “protein tyrosine kinase 7” and “colon carcinoma kinase 4” in text words; **(D)** Search keyword “PTK7 and cancer”; **(E)** Distribution of the count of studies on PTK7 in different cancer types; **(F)** Distribution of the count of studies on PTK7 in digestive system cancers.

Since its initial discovery in the last century, the number of studies on PTK7 has been increasing annually. A search on PubMed using keywords such as “Protein Tyrosine Kinase 7” and “Colon Carcinoma Kinase 4” reveals a growing number of relevant studies (**Figures 2A–C**). Prior to 2010, the number of studies remained in single digits. However, the number of studies has increased since 2014, leading to the first peak. Subsequent years show fluctuations but reach a second peak in 2019-2020.

In terms of studies on PTK7 and cancer, apart from colon carcinoma, the first report was on melanoma in 1997 (47). Over the 21st century, the number of studies on PTK7 in cancer has increased significantly, reflecting the importance of cancer research in biomedicine (**Figure 2D**). A considerable portion of PTK7-related studies focuses on various cancer types, with studies in digestive system cancers comprising the largest proportion (**Figure 2E**). Hematologic malignancies rank second, followed by lung cancer, particularly non-small cell lung cancer. The digestive system category includes research on colorectal cancer, which aligns with the historical context of PTK7’s discovery (**Figure 2F**). Moreover, studies on PTK7 in gastric and esophageal cancers are also prevalent. However, the number of studies on PTK7 in biliary tract cancer is limited due to lower incidence rates and challenges in obtaining pathological tissues. In contrast, research on PTK7 in breast cancer, as a single-organ cancer, ranks fourth in terms of study numbers.

Of course, the data presented here are based on incomplete statistics, but the research activity surrounding PTK7 in various cancer types seems to align with the incidence and research focus on specific cancer types, indicating that PTK7 has been extensively studied in a variety of cancers.

## 3 Roles of PTK7 in different cancer types

### 3.1 Digestive system cancer

**Figure 3:**
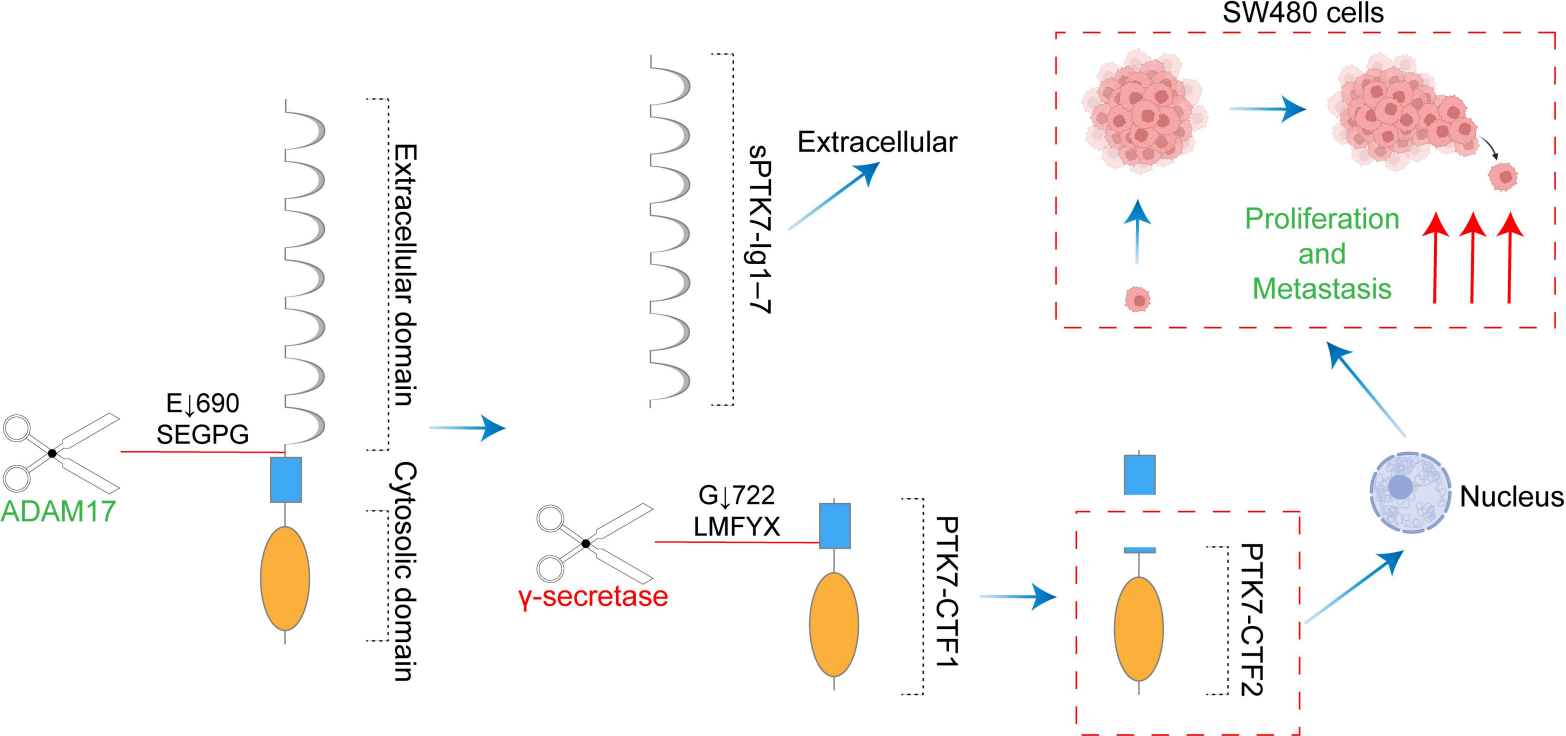
The cytosolic domain of PTK7 generated by sequential cleavage of ADAM17 and γ-secretase promotes colon cancer tumorigenesis. ADAM17 catalyzed the shedding of PTK7 into sPTK7-Ig1-7 and PTK7-CTF1. γ-secretase mediated further cleavage of PTK7-CTF1 to PTK7-CTF2. PTK7-CTF2 translocation to the nucleus promotes the proliferation and migration of SW480 cells.

**Figure 4:**
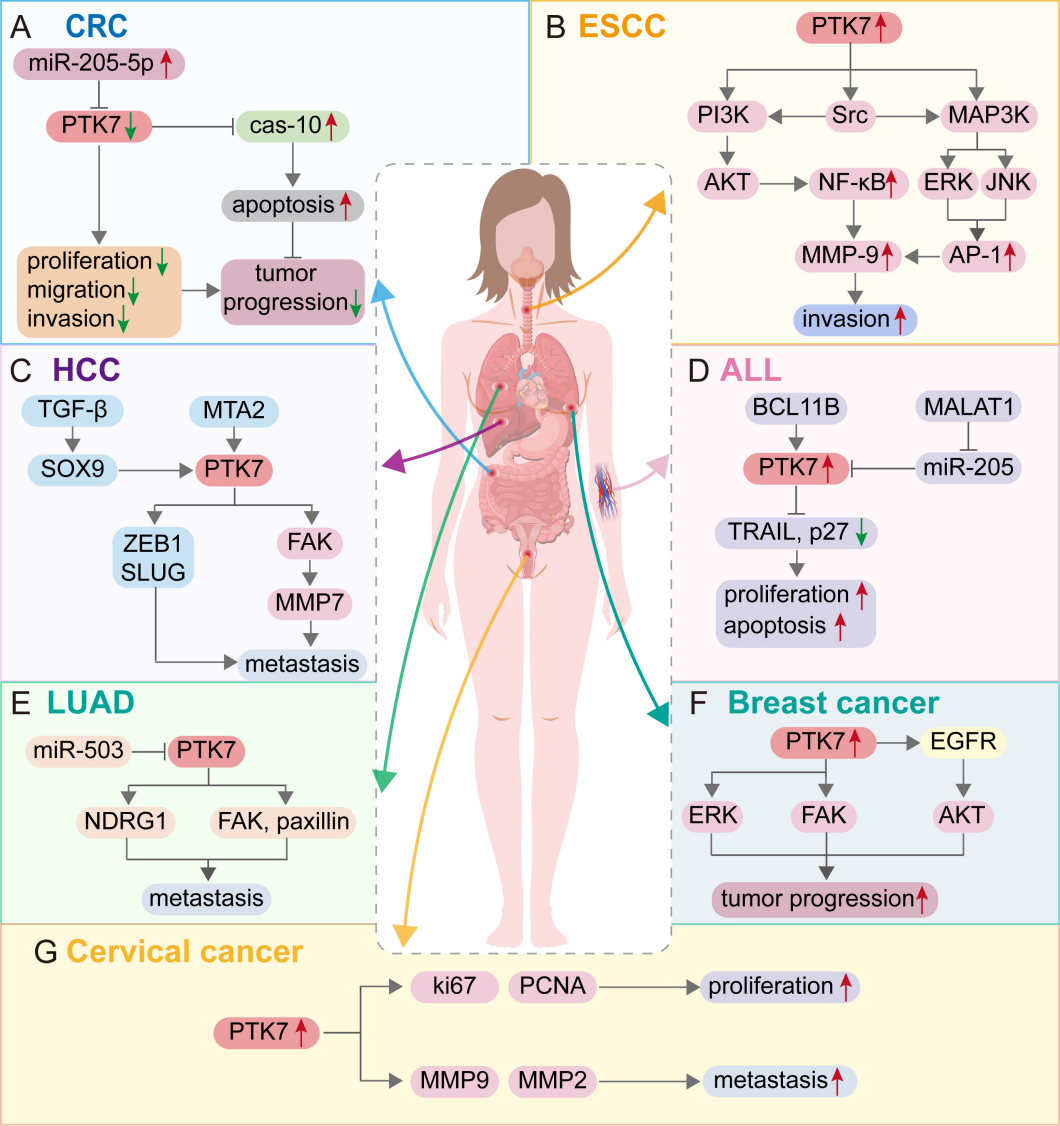
Molecular mechanism of PTK7 regulation of tumor progression in different cancer types. **(A)** Colon cancer; **(B)** Esophageal squamous cell carcinoma; **(C)** Hepatocellular carcinoma; **(D)** Acute lymphocytic leukemia; **(E)** Lung adenocarcinoma; **(F)** Breast cancer; **(G)** Cervical cancer. Red arrows represent the promotion of cancer progression, and green arrows represent the inhibition of cancer progression.

With the initial discovery of the complementary DNA encoding CCK-4 in colon cancer tissue, attention has been focused on this novel member of the RTK family. Although no significant expression of CCK-4 mRNA was observed in adult colon tissues, its expression in colon cancer-derived cell lines was found to be significantly different (3). The upregulation of PTK7 in CRC has been validated by multiple studies (41, 42). Mechanistically, it was initially identified that PTK7 signaling is regulated by SEMA6 and Plexin-A family members, impacting the PCP pathway through VANGL (48). Knocking down PTK7 inhibited cell proliferation and induced caspase-10-dependent apoptosis via the mitochondrial pathway (49). A subsequent study found that the cytosolic domain of PTK7, which could promote tumorigenesis, is generated by sequential cleavage of ADAM17 and γ-secretase. PTK7 is first shed into a form containing seven immunoglobulin-like loops (sPTK7-Ig1-7) and two C-terminal fragments (CTF). In this process, the shedding of PTK7 into sPTK7-Ig1-7 and PTK7-CTF1 is catalyzed by ADAM17, and further cleavage of PTK7-CTF1 into PTK7-CTF2, which is localized in the nucleus, can promote cell proliferation and migration and is mediated by the γ-secretase complex (50) (**Figure 3**). Besides, it has also been found that the transcription of PTK7 can be repressed by miR-205-5p (51). Thus, the miR-205-5p/PTK7/CASP10 axis is now a well-established important pathway for the involvement of PTK7 in CRC progression (**Figure 4A**). Several other potential indirect regulatory mechanisms of PTK7 in CRC have also been uncovered. For example, PTK7 was identified as a ligand for Macrophage Galactose-type Lectin (MGL) on the surface of CRC cells, implicating PTK7 in CRC immune evasion and tumor growth (52). Moreover, an organoid study found that APC mutant microsatellite-stable sporadic early-onset colorectal cancer (EOCRC) organoids exhibited high expression of PTK7 stem cell markers, suggesting a strong association between PTK7 and specific genomic features of APC mutant CRC (53). Interestingly, in normal organs, calcitriol was found to upregulate the expression of various stemness-related genes, including PTK7, whereas in tumor organs, calcitriol had minimal impact on PTK7 expression, indicating a loss of regulation by calcitriol in CRC (54). Lastly, emphasis is placed on the germline variant in PTK7. Recent research revealed that the PTK7^V354M^ variant increases PTK7 protein levels by potentially influencing protein stability and promotes cell proliferation, invasion, and migration. Furthermore, the PTK7^V354M^ variant inhibits p53, p21, and CREB gene transcription and protein expression, while increasing AKT protein expression. Interestingly, inhibition of AKT failed to rescue CREB expression in the presence of the PTK7^V354M^ variant. Additionally, the PTK7^V354M^ variant enhances cell cycle progression and upregulates Wnt downstream targets (55).

Studies on PTK7 in gastric cancer (GC) were reported in the early 21st century. A study confirmed through array comparative genomic hybridization that the PTK7 gene on chromosome 6p21 is overexpressed and amplified in GC (56). The amplification status of PTK7 was also reconfirmed in a subsequent study (57). However, over the past 20 years, few studies have examined the biological functions of PTK7 in GC. Does this imply that PTK7 is irrelevant in GC? Although the molecular mechanism of PTK7 involvement in GC remains unclear, it has been confirmed that PTK7 expression correlates with the prognosis of GC patients (58, 59). Thus, PTK7 is likely to play significant roles in the development of GC, and researchers in related fields still need to explore the hidden molecular mechanisms behind this.

Studies of PTK7 in esophageal cancer have mainly focused on esophageal squamous cell carcinoma (ESCC). Literature has confirmed that PTK7 expression is upregulated in ESCC (40, 60–63). It was demonstrated that PTK7 knockdown inhibited ESCC cell proliferation, migration, and invasion, and reduced the survival of 5-FU-treated cells. Mechanistically, knockdown of PTK7 significantly decreased the phosphorylation levels of Akt, Erk, JNK, p38 MAPK, and FAK, which may subsequently inhibit the proliferation, survival, migration, and invasion of ESCC cells (61). Additionally, PTK7 could enhance the invasive properties of cancer cells by upregulating MMP9 through activating AP-1 and NF-κB in ESCC (64). In detail, PTK7 initially activates the PI3K/AKT and Ras/MAPK pathways, leading to the activation of NF-κB by the PI3K-Akt signaling cascade and AP-1 by ERK and JNK. Subsequently, NF-κB and AP-1 transactivate MMP9, enhancing the invasive phenotype of ESCC cells (**Figure 4B**). Furthermore, NF-κB has been found to be crucial in mediating tumor radioresistance in ESCC through the NF-κB-dependent apoptosis pathway modulated by PTK7 (65). It has also been discovered that the migration of ESCC cells mediated by PTK7 may involve the inhibition of E-cadherin. Significant upregulation of major apoptosis regulators such as p53 and caspases was observed in siPTK7 cells, suggesting increased levels of apoptosis (62). However, PFTα seems to reverse the ESCC progression inhibited by PTK7 knockdown (63). Therefore, the interaction between PFTα and PTK7 appears to influence the fate of ESCC cells, warranting further investigation to identify rational therapeutic targets. Findings from the aforementioned *in vitro* experiments support the notion that PTK7 promotes ESCC progression. The effect of PTK7 on ESCC growth is not uniform, as recent research suggests that PTK7 may have a biphasic effect on tumorigenesis in ESCC. In ESCC cell lines with high PTK7 expression, overexpression of PTK7 inhibited cancer cell progression, in contrast to ESCC cell lines with lower PTK7 expression levels, leading to the hypothesis that ESCC cell malignancy may initially increase and then decrease with PTK7 expression (40). Controversially, the effect of PTK7 on TE-10 cells in this study is inconsistent with previous studies (61, 64). So, how exactly does PTK7 affect ESCC cells? A subsequent study demonstrated the ability of PTK7 to inhibit the development of ESCC in xenograft tumors (66). Meanwhile, the anti-PTK7 monoclonal antibodies exhibit anti-cancer activity both *in vivo* and *in vitro (* 67). Although the effect of PTK7 expression on ESCC is complex, in most cases, PTK7 is able to promote ESCC progression, and this regulatory mechanism is targetable in anticancer therapy.

In hepatocellular carcinoma (HCC), it was first shown that PTK7 is downregulated and that the mechanism of PTK7 silencing is associated with promoter hypermethylation (68). However, a study based on the TCGA-HCC cohort found that PTK7 was expressed at higher levels in HCC than in non-cancerous tissues (69). Similar results were obtained in subsequent studies (70, 71), which indicated that PTK7 expression was elevated in HCC. The conclusion of the low expression of PTK7 in HCC in previous studies may be due to limited sample size or population differences. Besides, CCNB1 mutant samples had lower PTK7 expression levels compared to CCNB1-wildtype samples. Mechanistically, PTK7 knockdown could inhibit cell migration and invasion. As a downstream molecule of MTA2, PTK7 can promote HCC metastasis through the FAK-MMP7 axis (70). Animal models also confirmed that PTK7 was positively correlated with the ability of HCC cells to extravasate into the lung. The prometastatic potential of PTK7 is mediated primarily by its secretory domain. Meanwhile, both PTK7 and its upstream regulator SOX9 were closely associated with HCC metastasis, and the accumulation of PTK7 was the result of SOX9-mediated activation of TGF-β signaling. In addition, the upregulation of PTK7 resulted in the enrichment of the EMT components SLUG and ZEB1, which also conferred PTK7’s prometastatic properties (71). However, this study did not find any evidence suggesting that activated Wnt signaling is responsible for the accumulation of PTK7 and promotes the metastasis of HCC, which deserves deeper investigation and reflection. The above two potential pathways of PTK7 involved in HCC metastasis are displayed in **Figure 4C**.

In another major type of primary liver cancer, intrahepatic cholangiocarcinoma (ICC), PTK7 was also upregulated. ICC cell lines with higher levels of PTK7 expression had enhanced proliferation, DNA synthesis, invasion, and migration, which could be inhibited by PTK7 knockdown. Meanwhile, PTK7 silencing induced apoptosis and also mediated a slight decrease in the expression levels of the cell cycle-related proteins CDK2, CDK4, CDK6, and cyclin D1, along with an increase in the expression levels of p16, p21, and p27, which may be a potential mechanism by which PTK7 silencing inhibits ICC progression. In addition, the association of PTK7 with the PCP pathway was also validated in ICC cells (72).

In conclusion, the upregulation and carcinogenic role of PTK7 in digestive system cancers have been confirmed, and the molecular mechanisms behind this have been initially elucidated (**Table 1**). How to target PTK7 in digestive system cancers is destined to become the focus of future research.

**Table 1:** Role of PTK7 in digestive system cancer.

Cancer type	Regulation	Mechanism	Ref
Colon cancer	Upregulated	Regulated by SEMA6 and Plexin-A family members, affects the PCP pathway through VANGL.	(48)
		Knockdown inhibits cell proliferation and induces caspase-10-dependent apoptosis via the mitochondrial pathway.	(49)
		The cytosolic domain of PTK7 generated by sequential cleavage of ADAM17 and γ-secretase promotes cell proliferation and migration.	(50)
		miR-205-5p inhibits cell proliferation, migration, and invasion and induces apoptosis by suppressing PTK7 transcription.	(51)
		Acts as an MGL-ligand on the surface of cancer cells and may mediate immune evasion and tumor growth.	(52)
		APC mutant microsatellite-stable EOCRC organoids had high PTK7 stem cell marker expression.	(53)
		PTK7 expression in CRC organoids is no longer regulated by calcitriol as in normal organoids.	(54)
		PTK7V354M variant increases PTK7 protein levels by potentially altering protein stability and promotes proliferation, invasion, and migration of CRC cells.	(55)
		PTK7V354M variant inhibits p53, p21 and CREB gene transcription and protein expression, increase AKT protein expression, increases cell cycle progression and up-regulates Wnt downstream targets.	
Gastric cancer	Overexpressed	Unknown.	
ESCC	Upregulated	Knockdown reduces the phosphorylation levels of Akt, Erk, JNK, p38 MAPK, and FAK, and may consequently inhibit cell proliferation, migration, and invasion and decrease the survival of 5-FU-treated ESCC cells.	(61)
		PTK7 increases ESCC cell invasiveness by upregulating MMP9 through the activation of AP-1 and NF-κB.	(64)
		PTK7 mediates tumor radioresistance by affecting NF-κB-dependent apoptosis.	(65)
		PTK7 inhibits E-cadherin to promote cell migration, and the knockdown of PTK7 upregulates p53 and caspases to promote apoptosis.	(62)
		Inhibition of ESCC cell progression by PTK7 knockdown can be reversed by PFTα.	(63)
		PTK7 expression can biphasically regulate ESCC progression.	(40)
HCC	Upregulated	PTK7 acts as a downstream molecule of MTA2 to promote HCC metastasis through the FAK-MMP7 axis.	(70)
		PTK7 expression was higher in CCNB1 wildtype HCC.	(71)
		SOX9-mediated activation of TGF-β leads to the accumulation of PTK7 and thus promotes HCC metastasis.	
		PTK7 leads to the enrichment of the EMT components SLUG and ZEB1, which mediate HCC metastasis.	
ICC	Upregulated	PTK7 expression promotes cell proliferation, DNA synthesis, invasion and migration.	(72)
		PTK7 silencing inhibits ICC progression, induces apoptosis, downregulates CDK2, CDK4, CDK6 and cyclin D1, and upregulates p16, p21 and p27.	

### 3.2 Hematologic malignancy

An earlier high-throughput analysis of genome-wide RTK expression in human cancers identifies PTK7 as overexpressed in acute myeloid leukemia (AML) (73). Moreover, PTK7 was more positively expressed in FAB AML M1, M2, and M6 compared to FAB AML M4 and M5. Meanwhile, in FAB AML M4, the expression of PTK7 was always restricted to the granulocytic committed blasts and poorly expressed in their monocytic counterparts. In all FAB subgroups, PTK7 positivity was higher in CD34+ and CD117+ cells and lower in CD11b+ and CD15+ cells. These findings suggest that PTK7 expression in AML is associated with different cytologic profiles. *In vitro* experiments exploring the roles of PTK7 showed that PTK7 promotes leukemia cell migration, cell survival, and resistance to anthracycline-induced apoptosis. The intracellular region is necessary for PTK7 to exert the above effects (74).

In chronic myeloid leukemia (CML), PTK7 expression was upregulated in leukemia cells compared to normal control cells and was suppressed by a dominant negative form of GAS2 (75). In T-ALL, nearly all cases expressed higher PTK7 levels than mature T cells in human bone marrow specimens (76, 77). PTK7 is a potential downstream target of BCL11B, and silencing BCL11B or PTK7 inhibits proliferation and induces apoptosis of ALL cells by upregulating TRAIL and p27 expression (77). Meanwhile, the expression of PTK7 in ALL was negatively affected by miR-205, and MALAT1 promotes proliferation and inhibits apoptosis of ALL cells through the miR-205-PTK7 axis (78) (**Figure 4D**).

Although there are many studies on PTK7 in hematologic malignancies, there are few studies exploring how PTK7 is involved in disease progression, and most of the current studies are directed toward therapeutic and clinical applications that target PTK7, which we will also address in more detail later.

### 3.3 Lung cancer

In lung adenocarcinoma, PTK7 was found to be overexpressed in cancer tissues compared to normal lung tissues, and the positivity of PTK7 expression was higher in cases with ALK mutations and lower in cases with EGFR mutations (37, 79). As for the role played by PTK7, it has been shown that PTK7 could enhance cancer cell adhesion by stabilizing NDRG1, and the PTK7-NDRG1 axis is recognized as a key factor in the development of resistance to AZD9291 (osimertinib) in lung adenocarcinoma cells (80). Meanwhile, PTK7, as a direct target gene of miR-503, could activate FAK and paxillin, thus regulating cytoskeletal dynamics to promote cancer cell invasion and migration, but PTK7 was not involved in miR-503-induced EMT (81) (**Figure 4E**).

In lung squamous cell carcinoma (LSCC), PTK7 unexpectedly exerts a cancer suppressor role, which is quite different from the role of PTK7 in lung adenocarcinoma and most other cancer types. Specifically, PTK7 is downregulated in LSCC, and overexpression of PTK7 inhibits LSCC cell proliferation, wound healing, and invasion, which may be mediated through the inhibition of ERK and AKT activation (82). Notably, the sample size involved in the analysis of PTK7 expression levels in this study was too small, whereas in the TCGA lung squamous cell carcinoma cohort, PTK7 is upregulated in cancer tissues. Given the much larger sample size of the TCGA cohort, we have to favor the conclusion that PTK7 is upregulated in LSCC.

### 3.4 Breast cancer

In breast cancer (BC), PTK7 expression levels are elevated, which is more pronounced in triple-negative breast cancer (TNBC) (39, 83, 84). Mechanistically, the knockdown of PTK7 reduces cell proliferation and inhibits the activation of FGFR1 and EGFR in BC cells (83). Besides, PTK7 upregulates EGFR/Akt signaling activation and is also associated with extracellular matrix organization and cytoskeleton remodeling (84) (**Figure 4F**). Meanwhile, the oncogenic properties of PTK7 have also been highlighted in TNBC. For example, *in vitro* experiments revealed that PTK7 was associated with TNBC cell growth, adhesion, migration, and invasion. *In vivo* experiments also verified that PTK7 is required for TNBC progression. The above effects exerted by PTK7 may be achieved by regulating the activation of ERK, Akt, FAK, and EGFR/Akt signaling (83, 84).

### 3.5 Melanoma

A late 20th-century study found that PTK7 mRNA expression was detected in only 54% of melanoma cell lines and 20% of melanoma biopsies. Similarly, PTK7 expression was absent in advanced cell lines screened from early cell lines that did express PTK7 mRNA (47). This seems to suggest that PTK7 expression is absent in metastatic melanoma. However, a recent study found that PTK7 expression was upregulated 67-fold in the metastatic murine B16-F10 melanoma cells compared to the murine melanoma B16-F1 cells (85). As can be seen, the expression of PTK7 in melanoma is still unclear, and its expression level may show significant changes with disease progression. Regarding the role played by PTK7 in melanoma, it has been shown that PTK7 is regulated by AMIGO2, and the two work together to promote tumor cell survival (86). The downregulation of PTK7 inhibits the formation and function of invadopodia, which is capable of promoting tumor metastasis and dissemination (87). Therefore, PTK7 may act more as an oncogene in melanoma, but whether and when PTK7 has cancer-inhibitory effects needs to be progressively explored in subsequent studies.

### 3.6 Gynecologic oncology

**Figure 5:**
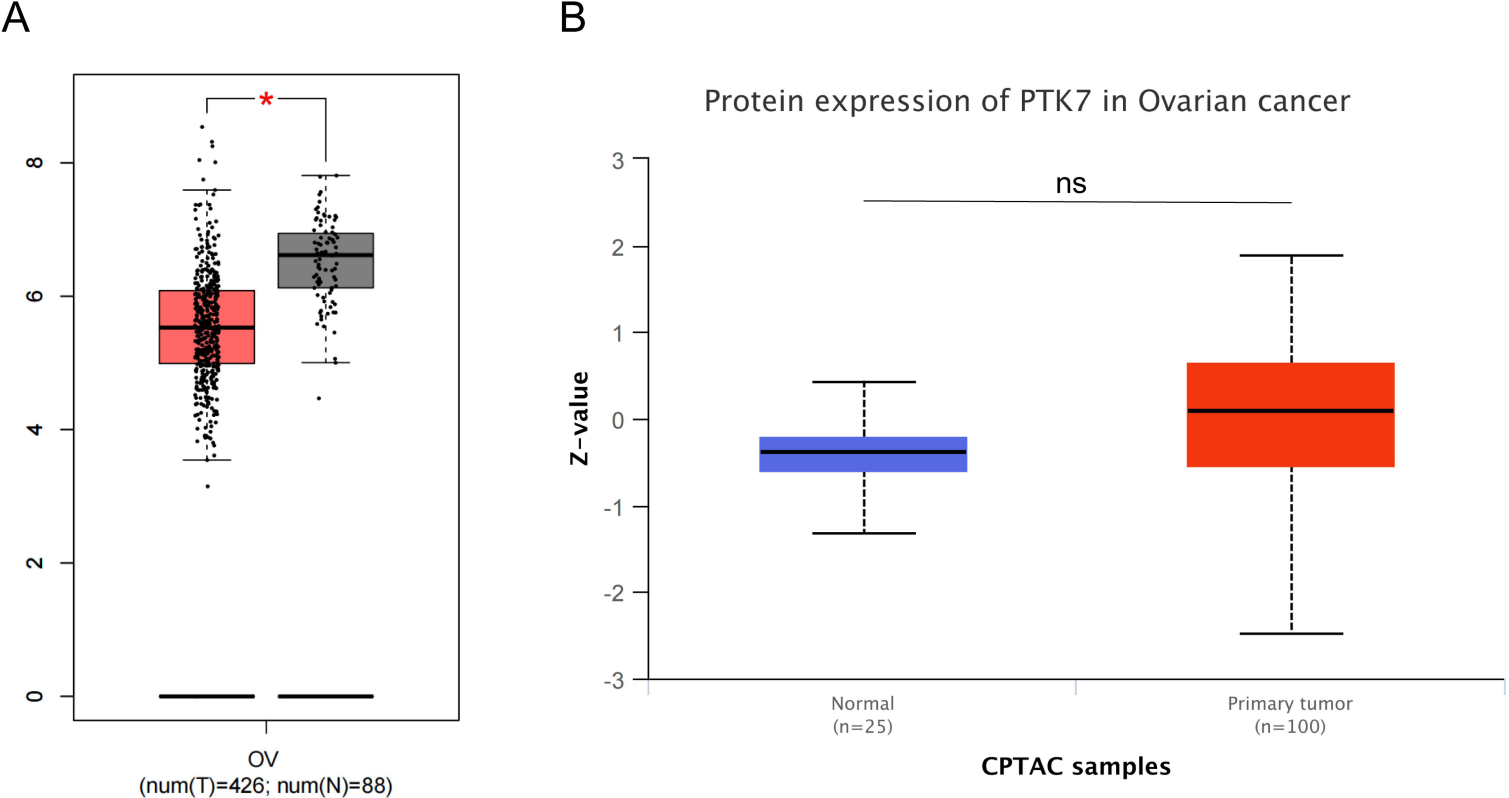
Expression of PTK7 in cancer tissues and normal tissues from public ovarian cancer cohorts. **(A)** PTK7 mRNA expression is downregulated in ovarian cancer tissues (GEPIA database); **(B)** Differences in PTK7 protein expression levels between ovarian cancer tissues and normal tissues were not significant (CPTAC database), *P < 0.05.

In ovarian cancer (OVCA), changes in PTK7 expression are still unknown. In the TCGA ovarian cancer cohort, the number of normal samples is too small to analyze. Analysis of the GEPIA database revealed that PTK7 mRNA expression was lower in ovarian cancer than in non-cancerous tissues after combining normal samples from the GTEx database with the TCGA cohort (**Figure 5A**). A study based on immunohistochemical staining analysis found significantly decreased expression levels of PTK7 from benign, intermediate, and malignant ovarian epithelial tumors and from normal controls to plasmacytoid carcinomas (88). The above results seem to indicate that PTK7 expression is downregulated in OV, but in fact, the sample sizes of normal tissues in both analyses were too small. However, PTK7 protein expression in the CPTAC ovarian cancer cohort did not differ between cancer tissues and noncancerous tissues (**Figure 5B**). Therefore, the changes in PTK7 expression in ovarian cancer remain unclear. A study has found that PTK7 expression is higher in the stem-A subgroup than in non-stem-A subgroup, suggesting that PTK7 is closely related to the stemness of ovarian cancer (89). It has also been found that SNCG and PP1γ are downstream targets of PTK7 in non-canonical Wnt5a signaling, and PTK7 regulates cell adhesion and Rho-GTPase signaling to sustain EMT and cellular plasticity (90).

In cervical cancer, overexpression of PTK7 has been confirmed by IHC assay. Mechanistically, *in vitro* experiments showed that PTK7 promotes cancer cell proliferation by modulating Ki67 and PCNA proteins. Meanwhile, PTK7 may promote cancer cell invasion by regulating MMP2 and MMP9 (91) (**Figure 4G**). The role of PTK7 in leukemia, lung cancer, breast cancer, melanoma, and gynecologic tumor is summarized in **Table 2**.

**Table 2:** Role of PTK7 in leukemia, lung cancer, breast cancer, melanoma, and gynecologic tumors.

Cancer type	Regulation	Mechanism	Ref
AML	Upregulated	PTK7 expression is mainly assigned to granulocytic lineage differentiation.	(74)
		PTK7 expression promotes cell migration, cell survival, and resistance to anthracycline-induced apoptosis.	
CML	Upregulated	PTK7 was suppressed by the dominant negative form of GAS2.	(75)
ALL	Upregulated	Downregulation of PTK7 via inhibition of the BCL11B pathway induces growth retardation and apoptosis of T-ALL cells.	(77)
		LncRNA‐MALAT1 regulates the proliferation and apoptosis of ALL cells via regulating the miR‐205‐PTK7 axis.	(78)
Lung adenocarcinoma	Upregulated	The positivity of PTK7 expression was higher in cases with ALK mutations and lower in cases with EGFR mutations.	(79)
		PTK7-NDRG1 axis regulates cell adhesion and mediates resistance to AZD9291.	(80)
		miR-503 regulates PTK7/FAK signaling to control the invasion and dissemination of cancer cells.	(81)
Lung SCC	Upregulated	PTK7 inhibits ERK and AKT activation, suppressing cell proliferation, wound healing, and invasion.	(82)
Breast cancer	Upregulated	PTK7 knockdown reduces cell proliferation and inhibits activation of FGFR1 and EGFR in BC cells.	(83)
		PTK7 knockdown decreases cell adhesion, migration, and invasion and inhibits the activation of ERK, Akt, and FAK in TNBC cells.	
		PTK7 upregulates EGFR/Akt signaling activation and is associated with extracellular matrix organization and cytoskeleton remodeling in BC cells.	(84)
		PTK7 promotes proliferation and migration in TNBC cells.	
		PTK7-deficiency inhibits TNBC growth *in vivo*.	
Melanoma	Unknown	PTK7 is regulated by AMIGO2 to promote melanoma cell survival.	(86)
		PTK7 downregulation inhibits the formation and function of invadopodia.	(87)
Ovarian cancer	Unknown	PTK7 is related to the stemness of ovarian cancer.	(89)
		SNCG and PP1γ are downstream targets of PTK7 in non-canonical Wnt5a signaling.	(90)
		PTK7 regulates cell adhesion and Rho-GTPase signaling to sustain EMT and cellular plasticity.	
Cervical cancer	Upregulated	PTK7 promotes cell proliferation by modulating Ki67 and PCNA.	(91)
		PTK7 may promote cell invasion by regulating MMP2 and MMP9.	

### 3.7 Urologic Tumors

In clear cell renal carcinoma (ccRCC), PTK7 expression was shown to be dowregulated, which is different from the changes in PTK7 expression in most solid tumors (92). Unfortunately, few studies have examined the role that PTK7 plays in ccRCC. In papillary renal cell carcinoma (PRCC), the second most common type of RCC, PTK7 expression was upregulated in some advanced cases. In detail, PTK7 expression was not significantly altered in localized-stage type 2 PRCC compared with normal samples. However, PTK7 expression was significantly upregulated in localized to advanced-stage type 2 PRCC, suggesting that PTK7 is associated with the progression of type 2 PRCC. At the same time, the upregulation of PTK7 in advanced-stage type 2 PRCC may be associated with PTK7 copy number gain (93).

Compared with benign prostatic hyperplasia tissues, PTK7 expression was upregulated in prostate cancer tissues (94). Meanwhile, PTK7 has a higher expression level in aggressive prostate cancer, suggesting that it may be related to cancer aggressiveness (95).

### 3.8 Head and neck cancer

In oral squamous cell carcinoma, the expression level of PTK7 was significantly upregulated (96, 97). Mechanistically, PTK7 knockdown inhibits OSCC cell viability, proliferation, invasion, and migration by downregulating DVL3 expression (96). PTK7 is also highly expressed in human thyroid cancer tissues, and PTK7 knockdown inhibits cell proliferation and promotes cell apoptosis *in vitro*. Meanwhile, PTK7 also has the property of promoting tumor growth in mice (98).

### 3.9 Brain tumor

In glioma, PTK7 was highly expressed in tumor tissues compared to nontumor brain tissues, especially in glioblastoma multiforme (GMB) tissues. A previous study showed that PTK7 expression was significantly positively correlated with the expression of CD44, a biomarker for the mesenchyma-like glioma subtype. Mechanistically, PTK7 knockdown attenuates tumor cell proliferation and impairs tumorigenic potential in CD44-high glioma cell lines. Besides, PTK7 induces anchorage-independent growth in normal astrocytes. The above effects are mediated by Id1, and the TGF-β/Smad signaling is an important pathway for PTK7 to regulate Id1 expression. *In vivo* experiments also confirmed that PTK7 depletion slowed tumor growth and prolonged the survival of tumor-bearing mice (99). Furthermore, a recent study found that PTK7 is a GPR133-binding protein in glioblastoma. PTK7-GPR133 binding takes place between their extracellular N-terminal domains, and PTK7 binding in trans increases GPR133 signaling. Meanwhile, knockdown of either GPR133 or PTK7 impairs glioblastoma tumorsphere formation. PTK7 has thus been identified as a positive allosteric modulator of GPR133 signaling in glioblastoma (100).

In medulloblastoma, PTK7 expression is also upregulated in tumor tissues (101). Thus, it is reasonable to speculate that PTK7 may also play important roles in medulloblastoma tumorigenesis. In neuroblastoma, PTK7 is highly and stably expressed on the tumor cell surface, but it demonstrates low expression in normal pediatric tissues. Despite the lack of evidence for the direct regulatory effects of PTK7 on neuroblastoma cell fate, anti-PTK7 CAR T cells could induce antigen-specific cytotoxicity against tumors and induce tumor cell death both *in vitro* and *in vivo (* 102). In atypical teratoid rhabdoid tumors (ATRT), PTK7 is also significantly overexpressed in tumor samples compared with normal brain samples. Inhibition of PTK7 has been shown to reduce the growth of ATRT cells (103).

### 3.10 Sarcoma

In liposarcoma, PTK7 was also upregulated. PTK7 knockdown inhibits cell proliferation and invasion and promotes cell apoptosis in LPS141 cell lines (104). In fibrosarcoma, a research team has explored the impact of PTK7 on the biological behavior of the HT1080 cells. They first found that shedding of the PTK7 ectodomain might contribute to cell invasion (105). Next, they demonstrated that both PTK7 expression and proteolysis, rather than the level of PTK7 alone, contribute to efficient directional cell motility and metastasis in HT1080 cells (106). However, PTK7 levels need to be tightly controlled to enable migration. In addition, they identified the anti-migratory effects of full-length membrane PTK7 in HT1080 cells, and this effect was associated with the down-regulation of multiple migration-related genes and activation of the Akt and c-Jun pathway (107). The role of PTK7 in urologic tumors, head and neck cancer, brain tumors, and sarcoma is summarized in **Table 3**.

**Table 3:** Role of PTK7 in urologic tumor, head and neck cancer, brain tumor, and sarcoma.

Cancer type	Regulation	Mechanism	Ref
CCRC	Downregulated	Unknown.	
PRCC	Upregulated	PTK7 copy gain leads to higher PTK7 expression in advanced-stage type 2 PRCC.	(93)
Prostate cancer	Upregulated	PTK7 is expressed at higher levels in aggressive prostate cancer.	(95)
OSCC	Upregulated	PTK7 knockdown inhibits cell viability, proliferation, invasion, and migration by downregulating DVL3.	(96)
Thyroid cancer	Upregulated	PTK7 knockdown inhibits cell proliferation and promotes cell apoptosis *in vitro*.	(98)
		PTK7 contributes to tumor growth in mice.	
Glioma	Upregulated	Higher PTK7 expression was consistent with higher CD44 expression in the mesenchyma-like glioma subtype.	(99)
		PTK7 regulates Id1 expression to promote cell proliferation and tumorigenesis, inhibit apoptosis of CD44-high gliomas, and induce anchorage-independent growth of normal astrocytes through the TGF-β/Smad signaling.	
		PTK7 depletion slows tumor growth and prolongs tumor-bearing mice survival *in vivo*.	
Glioblastoma	Upregulated	PTK7 is a GPR133-binding protein, and PTK7-GPR133 binding occurs between their extracellular N-terminal domains.	(100)
		PTK7 binding in trans increases GPR133 signaling.	
		Knockdown of PTK7 impairs glioblastoma tumorsphere formation.	
Medulloblastoma	Upregulated	Unknown.	
Neuroblastoma	Upregulated	Anti-PTK7 CAR T cells induce antigen-specific cytotoxicity against tumors.	(102)
		PTK7-targeting CAR T cells induce tumor cell death *in vitro* and *in vivo*.	
ATRT	Upregulated	PTK7 inhibition reduces tumor cell growth.	(103)
Liposarcoma	Upregulated	PTK7 knockdown inhibits cell proliferation and invasion and promotes cell apoptosis.	(104)
Fibrosarcoma	Unknown	Shedding of the PTK7 ectodomain might contribute to HT1080 cells invasion.	(105)
		Protein hydrolysis of PTK7 also contributes to efficient targeted cell motility and metastasis.	(106)
		Anti-migratory effects of full-length membrane PTK7 are associated with Akt and c-Jun pathway activation.	(107)

## 4 PTK7 as a prognostic biomarker for multiple cancer types

PTK7 expression correlates with the fate of cancer cells and then inevitably with the clinical outcome of cancer patients. Here, we review the findings of previous studies and comprehensively summarize the association between PTK7 and the prognosis of patients with different cancer types. In view of the fact that the expression of molecules involves multiple levels, such as transcriptome and proteome, and that different experimental methods may lead to inconsistent measurements, as well as the importance of statistical methods for the interpretation of the results of the survival analysis, we show the above methods together in **Table 4**.

**Table 4:** Prognostic significance of PTK7 in different cancer types.

Cancer type		Prognosis	Follow-up	Data source	Statistical method	Ref
Digestive system	Colon cancer	Poor	MFS	IHC	uni-Cox	(41)
		Favorable	OS	IHC	log-rank test, multi-Cox	(42)
	Gastric cancer	Favorable	OS	IHC	log-rank test, multi-Cox	(58)
			DFS			
			OS	IHC	log-rank test, multi-Cox	(59)
			DFS		log-rank test	
	ESCC	Poor	OS	IHC	multi-Cox	(61)
	HCC	Poor	OS	RNA-seq	multi-Cox	(69)
			OS	RNA-seq	log-rank test	(70)
			OS	RNA-seq, IHC	log-rank test	(71)
	ICC	Poor	DFS	IHC	log-rank test, multi-Cox	(72)
			OS	IHC	log-rank test, multi-Cox	
Hematology	AML	Poor	LFS	Flow cytometry	multi-Cox	(74)
			OS			
Lung	Lung Adenocarcinoma	Favorable	OS	RT-PCR	multi-Cox	(108)
Breast	Breast cancer	Poor	DFS	RT-PCR (LN)	log-rank test	(39)
		Poor	DFS	RT-PCR	log-rank test	(109)
			DFS	RT-PCR (LN)	log-rank test, uni-Cox	
	TNBC	Poor	OS	IHC	log-rank test	(84)
			RFS	RNA-seq	log-rank test	
Ggynecology	Serous ovarian cancer	Favorable	OS	IHC	log-rank test	(88)
Uurinary system	Prostate cancer	Poor	RFS	IHC	log-rank test, multi-Cox	(94)
			OS		multi-Cox	
	PRCC	Poor	OS	Copy number (gain)	log-rank test	(93)
	Adrenocortical Cancer	Poor	PFS	RNA-seq (ENST00000489707.5)	multi-Cox	(110)
			DSS			
Head and neck	OSCC	Poor	OS	IHC	log-rank test, uni-Cox	(97)
Brain	Glioblastoma	Poor	OS	RNA-seq	log-rank test	(99)
Mesenchymal tissue	Liposarcomagenesis	Favorable	DRFS	Gene microarray	multi-Cox	(104)

MFS, metastasis free survival; DFS, disease free survival; LFS, leukemia free survival; RFS, recurrence free survival; PFS, progression free survival; DSS, disease specific survival; DRFS, distant recurrence free survival; LN, lymph node.

In solid tumors of the digestive system, PTK7, although showing consistent overexpression, appears to have a differential impact on patient prognosis. In colon cancer, PTK7 expression implies poor metastasis-free survival (MFS) (41). However, another study concluded that PTK7 expression was associated with better OS (42). The quantification of PTK7 expression levels in both studies was based on IHC, and in addition to the effect of sample size on the conclusions, the correction of confounding factors by multivariate COX analysis may be one of the explanations for the almost contradictory conclusions of the two studies. In gastric cancer, although studies have shown that PTK7 is overexpressed, both studies confirmed that its expression is associated with better OS and disease-free survival (DFS) (58, 59), which suggests that PTK7 may be a double-edged sword in gastric cancer treatment. In ESCC, a study based on IHC found PTK7 to be an independent risk factor for OS in patients (61). Both PTK7 mRNA and protein expression suggest poor OS in HCC patients (69–71). PTK7 protein expression suggests poor OS and DFS in ICC patients (72).

In lung adenocarcinoma and serous ovarian cancer, PTK7 expression also correlated with better OS (88, 108). In liposarcomagenesis, PTK7 expression was associated with better distant recurrence-free survival (DRFS) (104). In addition to this, PTK7 expression in most other solid tumors is associated with poor prognosis. For example, in breast cancer patients not receiving chemotherapy, PTK7 mRNA expression in the primary tumor was associated with poor DFS (109). In TNBC, PTK7 expression implies poor OS and recurrence free survival (RFS) (84). Although this difference was not found in the whole population, it just suggests that PTK7 may be associated with chemosensitivity in breast cancer patients. Besides, PTK7 expression in lymph node tissues was also associated with poor DFS (39, 109). In prostate cancer, PTK7 protein can act as an independent risk factor for OS and RFS (94). In PRCC, although there is no evidence directly demonstrating that PTK7 expression is associated with poor prognosis, PTK7 copy number gain is associated with poor OS, and PTK7 gain samples tend to have higher PTK7 expression levels (93). It is also unclear whether PTK7 expression has prognostic significance in adrenocortical carcinoma, but the expression of ENST00000489707.5, a preferred alternative splicing variant of PTK7, is an independent risk factor for disease-specific survival (DSS) and progression-free survival (PFS) (110). As in most solid tumors, in oral squamous cell carcinoma and glioblastoma, PTK7 expression also implies poor OS (97, 99).

The prognostic value of PTK7 is not only in solid tumors, but also in acute myeloid leukemia, where its expression is an independent risk factor for leukemia free survival (LFS) and OS in patients (74). It can be seen that PTK7 is indeed a key molecule in the development of cancers, and its role not only involves the molecular mechanism of cancer progression but also is able to directly affect the survival of patients, which further confirms the value of PTK7 application in the clinical management of cancer.

## 5 PTK7 expression correlates with clinicopathologic characteristics

The malignant behavior of cancer driven by PTK7 is reflected not only in its impact on patient survival but also in the association of its expression with clinicopathological characteristics (**Table 5**). Although clinicopathological characteristics are always closely related to patient prognosis, the link between them and PTK7 can, to some extent, guide the individualized treatment of cancer, such as neoadjuvant therapy, conversion therapy, postoperative adjuvant therapy, etc.

**Table 5:** Correlation between PTK7 expression and clinicopathologic parameters.

Cancer type		Clinical significance	Data source	Ref
Digestive system	Colon cancer	well differentiation	IHC	(42)
		less LN metastasis		
		less distant metastasis		
		early TNM Stage		
	Gastric cancer	well differentiation	IHC	(58)
	HCC	late TNM Stage	RNA-seq	(69)
		late TNM Stage	RNA-seq	(70)
		poor differentiation		
		late TNM Stage	RNA-seq, gene microarray, IHC	(71)
		high risk of metastasis	gene microarray	
Hematology	AML	highly expressed in FAB M0,M1,M2,M3	flow cytometry	(74)
		poorly expressed in FAB M4,M5		
		associated with CD34 and CD117		
		lower white blood cell count		
		lower frequency of extramedullary disease		
Lung	Lung Adenocarcinoma	more LN metastasis	IHC	(79)
		more ALK mutation		
		less EGFR mutation		
Breast	Breast cancer	more LN metastasis	RT-PCR,IHC (LN)	(39)
		more LN metastasis	RT-PCR,IHC (LN)	(109)
	TNBC	highly expressed in TNBC subtype	IHC, gene microarray	(84)
		late TNM Stage	IHC	
		more LN metastasis		
Ggynecology	Epithelial ovarian cancer	highly expressed in type I tumors	IHC	(88)
		early clinical stage	IHC (borderline serous carcinoma)	
		less distant metastasis		
		early clinical stage	IHC (serous cancer)	
		lower Who’s grade		
		lower MDACC’s grade		
Uurinary system	Prostate cancer	more LN metastasis	IHC	(94)
		high frequency of seminal vesicle invasion		
		late clinical stage		
		high level of preoperative PSA		
		high Gleason Score		
		high frequency of angiolymphatic invasion		
		high risk of biochemical recurrence		
		high Gleason Score	IHC	(95)
Head and neck	OSCC	late TNM Stage	IHC	(111)
		poor differentiation		
		more LN metastasis	IHC	(97)
		poor differentiation		
		high YK class		
	Thyroid cancer	late TNM Stage	IHC	(98)
		high frequency of intraglandular dissemination		

LN, lymph node.

A previous study found that PTK7 was not only associated with better OS in colon cancer but also with good tumor differentiation, less lymph node metastasis and distant metastasis, and early TNM stage (42). Although this study contradicts another study regarding the effect of PTK7 on the prognosis of colon cancer (41), the association of PTK7 with clinicopathological characteristics seems to provide evidence for this. In gastric cancer, PTK7 expression was found to be associated with tumor differentiation as well (58). Given the cancer promoting properties of PTK7 in hepatocellular carcinoma, its expression has also been repeatedly confirmed to correlate with late TNM stage (69–71). Also, its expression was associated with poor tumor differentiation and high risk of metastasis (70, 71). Another controversial finding was in the field of lung adenocarcinoma, where it was found that PTK7 expression correlates with lymph node metastasis, and that high PTK7 expression implies a higher rate of ALK mutation and a lower rate of EGFR mutation (79). Lymph node metastasis clearly contradicts the better OS described previously (108). Differently, the two studies were analyzed from the protein level and mRNA level using IHC and RT-PCR, respectively, while the prognostic significance of PTK7 was tapped during the construction of prognostic gene signature by multivariate COX analysis. The research perspectives of the two studies were different, so the findings cannot be directly compared.

In breast cancer, several studies have confirmed that PTK7 expression in lymph node tissues is strongly associated with lymph node metastasis (39, 84, 109). Meanwhile, significantly high expression of PTK7 correlated with the TNBC subtype and suggested advanced TNBC (84). In epithelial ovarian cancer, PTK7 is highly expressed in type I tumors. PTK7 expression is associated with early clinical stage and less distant metastasis in borderline serous carcinomas. As for serous cancer, PTK7 expression was associated with lower Who’s grade and MDACC’s grade in addition to tumor clinical stage (88). In prostate cancer, higher PTK7 expression means more lymph node metastasis, later tumor stage, higher frequency of seminal vesicle invasion and angiolymphatic invasion, higher levels of preoperative PSA, higher risk of biochemical recurrence (94), and higher Gleason score (94, 95). It can be seen that the initial risk grouping of prostate cancer patients can be done directly based on the expression level of PTK7. The correlation between PTK7 and clinicopathological characteristics of head and neck cancer was also consistent with its correlation with patient prognosis. In oral squamous cell carcinoma, higher PTK7 expression implies not only a later TNM stage (111), more lymph node metastasis (97), and poorer differentiation (97, 111), but also a higher risk of YK classification (97). As for thyroid cancer, PTK7 expression is associated with advanced TNM stage and a high frequency of intraglandular dissemination (98).

PTK7 also correlates significantly with clinical parameters in acute myeloid leukemia. First, PTK7 expression was associated with FAB classification. In detail, PTK7 is highly expressed in FAB-AML M0, M1, M2 and M3, and poorly expressed in FAB-AML M4 and M5. Then, the occurrence rates of CD34+ and CD117+ were higher and CD11b+ and CD15+ were lower in PTK7+ cases. Finally, PTK7+ AMLs were associated with a significantly lower white blood cell count at diagnosis and with a lower frequency of extramedullary disease. However, this correlation is based on univariate analysis only, and subsequent removal of the effects of confounders is still needed to further confirm the finding (74). Taken together, the above findings not only explain the prognostic significance of PTK7, but also provide guidance for the clinical management of cancer patients.

## 6 Cross-talk between PTK7 and cancer therapy

Given the outstanding research results of PTK7 in the field of cancer, targeting PTK7 has long been a hot research topic in the field of cancer treatment. Unfortunately, as of now, no PTK7-targeted drugs have been approved for marketing in the world. Currently, PTK7 drugs in development include ADC, aptamer, CAR-T, mAb and other types, but only a few drugs have entered the clinic, and all of them are in clinical phase I. Most of the drugs are in the preclinical or biological testing stage. Here, we summarize in chronological order the results of recent research on PTK7 in cancer therapy (**Table 6**).

**Table 6:** Research progress of PTK7 in anticancer therapy.

Year	Category	Drug abbreviation	Therapeutic performance	Phase	Ref
2010	aptamer	sgc8-daunorubicin complex	Able to specifically deliver and internalize daunorubicin to PTK7+ ALL-T cells	Preclinical-*In vitro* and *in vivo*	(112)
2011	aptamer	Dox: sgc8/hp-Au NPs	Light-induced release of doxorubicin leads to more effective killing of PTK7+ cancer cells with fewer side effects.	Preclinical-*In vitro* and *in vivo*	(113)
2017	ADC	PF-06647020 (Cofetuzumab pelidotin)	PF-06647020 elicited potent antitumor activity, reduced TIC frequency in low-passage PDXs, and exhibited a favorable safety profile in nonhuman primates.	Preclinical-*In vitro* and *in vivo*	(114)
2019	versatile polyion complex	APSP/HA	APSP/HA owes its capabilities to prolonged circulation time, elevated tumor penetration, the facilitation of cancer stem cell targeting, and increased intracellular triphenylphosphine-docetaxel and miR-31 accumulation.	Preclinical-*In vitro* and *in vivo*	(115)
2019	aptamer (nanogel)	Dox-loaded DNA-protein hybrid nanogel	Capable of selective uptake by target cell lines through a receptor-mediated endocytosis mechanism and delivery of doxorubicin within the cell line.	Preclinical-*In vitro*	(116)
2019	aptamer (DNA tetrahedral nanostructure)	s-TDN: DOX	s-TDN: DOX exhibited enhanced cytotoxicity against PTK+ CCRF-CEM cells, with a minor effect against PTK7- Ramos cells.	Preclinical-*In vitro*	(117)
2019	aptamer (DNA nanoflowers)	Sgc8-NFs-Fc/Dox	Sgc8-NFs-Fc nanocarriers are size and functionally tunable, self-degradable, and have on-demand drug release kinetics upon H2O2 stimulation.	Preclinical-*In vitro* and *in vivo*	(118)
2020	aptamer-Pyropheophorbide conjugates	APCs	APCs bind specifically to PTK7+ cancer cells and penetrate efficiently into the tumor interior, exhibiting favorable phototoxicity to target tumor cells.	Preclinical-*In vitro*	(119)
2020	aptamer (DNA nanostructure)	PA/PDN	Photosensitizer/Doxorubicin/Antisense oligonucleotide-loaded PA/PDN showed greater photodynamic activity, higher anticancer activity, and selective reduction of target proteins in PTK7+ cancer cells.	Preclinical-*In vitro*	(120)
2020	aptamer (Evans Blue-modified Sgc8)	EB-Sgc8/HSA complex	EB-Sgc8/HSA complex exhibited prolonged blood half-life and increased tumor accumulation.	Preclinical-*In vitro* and *in vivo*	(121)
2020	CAR-T	PTK7 CAR-T cells	Exhibited anticancer activity but showed transient weight loss and other potential toxicities.	Preclinical-*In vitro* and *in vivo*	(128)
2021	ADC	PF-06647020 (Cofetuzumab pelidotin)	PF-06647020 showed therapeutic activity in patients with an acceptable incidence of TRAEs, supporting further clinical evaluation.	Clinical-Phase I	(45)
2021	CAR-T	PTK7 CAR2-T cells	Systemic delivery of PTK7-CAR2 T cells significantly prevented tumor growth and prolonged the overall survival of mice without detectable tumor off-target toxicity.	Preclinical-*In vitro* and *in vivo*	(129)
2022	aptamer-Gemcitabine conjugate	PTK7-GEMs	PTK7-GEMs specifically bind to cancer cells dependent on the expression level of PTK7 and showed stronger anti-tumor efficacy and excellent biosafety in tumor xenograft mice models.	Preclinical-*In vitro* and *in vivo*	(122)
2022	ADC + PI3K/mTOR inhibitor	Cofetuzumab Pelidotin + Gedatolisib	The combination of Cofetuzumab Pelidotin + Gedatolisib showed good clinical tolerability and preliminary anticancer activity.	Clinical-Phase I	(46)
2022	mAb	Cofetuzumab	A combination of other targeted or chemotherapeutic agents with Cofetuzumab may increase the pharmacological efficacy.	Preclinical-*In vitro*	(90)
2022	mAb	mAb-32 and mAb-43	PTK7 mAbs exhibit anticancer activity and reduce PTK7 levels *in vivo* and *in vitro*.	Preclinical-*In vitro* and *in vivo*	(67)
2022	PTK7/β-Catenin Inhibitors	Compounds 01065, 03653 and their analogs	Small molecule inhibitors of the PTK7/β-Catenin interaction inhibit CRC cell growth *in vitro*.	Preclinical-*In vitro*	(127)
2023	aptamer-Dasatinib hybrids	Sgc8-c-carb-da	Sgc8-c-carb-da was able to release dasatinib at endosomal-pH, and *in vitro* experiments revealed a higher cytotoxic effect of Sgc8-c-carb-da than dasatinib.	Preclinical-*In vitro*	(123)
2023	ADC	PF-06647020 (Cofetuzumab pelidotin)	NSCLC patients had a limited overall ORR of 19.6%, whereas the non-SCC EGFR WT subgroup had an ORR of 30%.	Clinical-Phase I	(124)
2023	ADC (anti-PTK7 x TROP2 bispecific)	BCG033	BCG033 candidates showed potent anti-tumor activity in several PTK7/TROP2 co-expressing cell line-derived xenografts.	Preclinical-*In vitro*	(125)
2023	ADC	MTX-13	MTX-13 demonstrated anticancer activity and the ability to overcome tumor resistance and has the potential to outperform PF-06647020 in clinical trials.	Preclinical-*In vitro* and *in vivo*	(44)
2023	ADC	PRO1107	PRO1107 is superior to PF-06647020 in terms of antitumor activity and tolerability and also has a bystander effect.	Clinical-Phase I	(126)
2023	CAR-T	TREM1/DAP12-based PTK7 CAR-T cells	Exhibited potent cytotoxicity against PTK7+ ovarian cancer cells.	Preclinical-*In vitro* and *in vivo*	(130)
2023	CAR-T	PTK7 CAR-T cells	Inhibits neuroblastoma progression without significant toxicity.	Preclinical-*In vitro* and *in vivo*	(102)
2023	CAR-aptamers	CAR-bc-ap	CAR-aptamers enable traceless enrichment and monitoring of CAR-positive cells and overcome tumor immune escape.	Preclinical-*In vitro* and *in vivo*	(131)
2023	aptamer-antibody complexes	20S-sgc8-OKT3	20S-sgc8-OKT3 enhances the antitumor effect of T cells by stimulating activation and cytokine secretion.	Preclinical-*In vitro*	(132)

In 2010, a study constructed aptamer-drug complexes via sgc8, an aptamer of PTK7, which resulted in the simple and efficient delivery of daunorubicin to PTK7+ ALL T cells. This avoided cytotoxic effects on PTK7- cells without decreasing the anticancer activity (112). A subsequent study designed a smart drug carrier, an aptamer/hairpin DNA-gold nanoparticle (Dox:sgc8c/hp-Au NP) conjugate for targeted delivery of drugs to kill cancer cells more efficiently and with fewer side effects by light-induced release of doxorubicin (113). Subsequently, the first PTK7-targeted ADC, PF-06647020, was designed. Comprehensive *in vitro* and *in vivo* experiments in TNBC, OVCA, and NSCLC demonstrated that PF-06647020 elicited potent antitumor activity, reduced TIC frequency in low-passage PDXs, and exhibited a favorable safety profile in nonhuman primates (114). Given the outstanding therapeutic potential of PF-06647020, it has also successfully entered clinical trials. Since then, more and more PTK7-based therapeutic modalities have been explored, including a versatile polyion complex called APSP/HA based on PTK7 antibody mediated active targetability. APSP/HA owes its capabilities to prolonged circulation time, elevated tumor penetration, the facilitation of lung cancer stem cell targeting and increased intracellular triphenylphosphine-docetaxel (TD) and miR-31 accumulation. TD induces apoptosis through a mitochondria pathway, whereas miR-31 modulates the MET-PI3K-Akt signaling pathway to exert the ability to eliminate CSCs (115). Besides, a study designed an aptamer-decorated nanogel, which is selectively taken up by CCRF-CEM and HeLa cells through a receptor-mediated endocytosis mechanism and delivers doxorubicin within the target cell lines, thereby exerting an anticancer effect (116). In the same period, another aptamer-targeted DNA nanostructure with doxorubicin (s-TDN: DOX) was also designed. It acts similarly to that of the aforementioned nanogel and exhibits enhanced cytotoxicity against PTK+ CCRF-CEM cells, with a minor effect against PTK7- Ramos cells (117). In addition, a class of bioinspired, size-controllable, and self-degradable cancer-targeting DNA nanoflowers (NFs) has also been designed. Sgc8-NFs-Fc nanocarriers have the advantages of adjustable size and functionality, self-degradability, and on-demand drug release under H2O2 stimulation (118).

PTK7-targeting aptamer has also made advances in the field of cancer photodynamic therapy (PDT). The molecular probe aptame-Pyropheophorbide conjugates (APCs) bind specifically to PTK7+ cancer cells and penetrate efficiently into the tumor interior, exhibiting favorable phototoxicity to target tumor cells (119). A study reported various therapeutic cargo-loadable DNA nanostructures that showed greater photodynamic activity, higher anticancer activity, and selective reduction of target proteins in PTK7+ cancer cell lines (120). Besides, to improve the targeted delivery performance of aptamers, prolonged blood circulation is an effective strategy. A study integrated an Evans Blue-modified Sgc8 (EB-Sgc8) with human serum albumin (HSA) to fabricate more effective anti-cancer drugs. EB-Sgc8/HSA complex did exhibit prolonged blood half-life and increased tumor accumulation (121). In bladder cancer, PTK7 aptamer-Gemcitabine conjugate (PTK7-GEMs) specifically bind to cancer cells dependent on the expression levels of PTK7 and PTK7-GEMs showed more robust anti-tumor efficacy and excellent biosafety in tumor xenograft mice models (122). Recently, another aptamer-drug conjugate, Sgc8-c-dasatinib hybrids, has also been developed to improve the therapeutic efficiency of lymphoma. Sgc8-c-carb-da, one of the best characterized Sgc8-c-dasatinib hybrids, was capable of releasing dasatinib at an endosomal-pH, and *in vitro* experiments revealed a higher cytotoxic effect of Sgc8-c-carb-da than dasatinib (123). It is evident that the aptamer is an important bridge between PTK7 and cancer therapy and was once a hot research topic across multiple disciplines.

In 2021, results from the first-in-human study of PF-06647020 in advanced solid tumors were announced. PF-06647020 showed therapeutic activity in patients with an acceptable incidence of TRAEs, supporting further clinical evaluation (45). In February 2020, the clinical Phase Ib trial of Cofetuzumab Pelidotin was initiated to explore its efficacy and safety in patients with PTK7+ recurrent NSCLC. The preliminary data was published at the European Society for Medical Oncology(ESMO) congress in 2023. NSCLC patients (n=56) had a limited overall ORR of 19.6%, whereas the Non-SCC EGFR WT subgroup had an ORR of 30% and an mPFS of 5.5 months. However, the low ORR response rate in patients with SCC, as well as the non-SCC EGFR mutant subgroup, may be the reason why the subsequent development of Cofetuzumab Pelidotin was terminated (124). However, a concurrent phase I safety study of Gedatolisib (pan-class I isoform PI3K/mTOR inhibitor) plus Cofetuzumab Pelidotin in patients with metastatic TNBC or ER-low (ER and PgR <5%, HER2-) breast cancer showed good clinical tolerability and preliminary anticancer activity, which makes further large sample size studies relevant (46). In addition to PF-06647020, there are several other PTK7-directed ADCs currently in the preclinical phase. BCG033, an anti-PTK7 x TROP2 bispecific ADC, has shown potent anti-tumor activity in several PTK7/TROP2 co-expressing cell line-derived xenografts (125). MTX-13, a novel PTK7-directed ADC, has demonstrated anticancer activity and the ability to overcome tumor resistance in pan-PTK7+ tumors and has the potential to outperform PF-06647020 in clinical trials (44). PRO1107, a recently approved PTK7-directed ADC, has just initiated its clinical phase 1/2 trial. Preclinical data proved that PRO1107 is superior to PF-06647020 in terms of antitumor activity and tolerability and also has a bystander effect (126). In summary, PTK7 has become a hot target for ADC drug development in recent years.

In addition, monoclonal antibodies (mAbs) and small molecule inhibitors of PTK7 have also demonstrated anticancer activity. A study found that the combination of other targeted or chemotherapeutic agents to anti-PTK7 mAb may increase the pharmacological efficacy compared to single-agent treatment in ovarian cancer (90). In ESCC, PTK7 mAbs exhibit anticancer activity and reduce PTK7 levels *in vivo* and *in vitro (* 67). In addition, small molecule inhibitors of the PTK7/β-Catenin interaction have been found to inhibit CRC cell growth *in vitro (* 127). In recent years, immunotherapy has gradually become a mainstream option for the systemic treatment of cancers. In 2020, a study confirmed the allogeneic anti-PTK7 CAR-T cells could exhibit anticancer properties both *in vitro* and in immunocompromised mouse xenograft models of cancer. However, consistent with all xenograft models studied, a drop in body weight was observed shortly after injection, from which all mice rapidly recovered to above baseline. Meanwhile, latent toxicity that was more variable in the animal experiments was also observed (128). A subsequent study constructed three different PTK7-specific CARs (PTK7-CAR1/2/3) and found that systemic delivery of PTK7-CAR2 T Cells significantly prevented tumor growth and prolonged overall survival of mice without detectable tumor off-target toxicity (129). Similar therapeutic properties have also been demonstrated in neuroblastoma (102). In ovarian cancer, PTK7 CAR-T cells based on TREM1/DAP12 signaling exhibited potent cytotoxicity against PTK7+ cancer cells (130). Because of this, the therapeutic potential of PTK7 aptamer and PTK7 CAR-T cells has enabled the exploration of more effective therapeutic strategies. CAR-specific binding aptamers (CAR-ap) enriched CAR-T cells were found to indeed possess enhanced antitumor activity *in vitro* and *in vivo*. Meanwhile, CAR-ap could monitor the expansion of CAR-T cells *in vivo*. Subsequently, a CAR-ap-based bispecific circular aptamer (CAR-bc-ap) is constructed by linking CAR-ap with Sgc8. The CAR-bc-ap could overcome immune escape by retargeting CAR-T Cells to tumors *in vitro* and *in vivo*, which makes it a potential new strategy for cancer treatment (131). Besides, a novel aptamer-antibody complex, 20S-sgc8-OKT3, was found to enhance the antitumor effect of T cells by stimulating activation and cytokine secretion (132). As expected, the link between PTK7 aptamers and T cell-based immunotherapy has thus been constructed.

PTK7 also has the potential to be a biomarker used for efficacy prediction prior to cancer treatment (**Table 7**). A single-center retrospective study found that among TNBC patients treated with anthracycline only, PTK7- cases had longer DFS than PTK7+ cases (38). Another study found that chemotherapy regimens other than anthracycline-based may benefit breast cancer patients with high LNT PTK7 expression (109). In colorectal cancer, APC mutant organoids are characterized by upregulated PTK7 expression, suggesting that PTK7 targeting may be beneficial for APC mutant cases (53). In addition, PTK7 may be used as a single biomarker for predicting the efficacy of anti-PD-1 treatment in patients with metastatic NSCLC (133). In summary, PTK7 can not only be a potential target for cancer therapy but also a biomarker for predicting the efficacy of chemotherapy and immunotherapy.

**Table 7:** Advances in PTK7 in predicting response to anticancer therapy.

Year	Cancer type	Clinical significance	Confidence	Ref
2013	TNBC	In patients treated with anthracycline only, DFS was longer in PTK7- cases than in PTK7+ cases.	single-center retrospective study (n=133)	(38)
2014	Breast cancer	Chemotherapy regimens other than anthracycline-based may benefit in patients with high LNT PTK7 expression.	single-center retrospective study (n=117)	(109)
2020	Colorectal cancer	APC mutant colon cancer organoids are characterized by upregulated PTK7 expression, suggesting that PTK7 targeting may be beneficial for APC mutant colorectal cancer patients.	exome and transcriptome analysis	(53)
2021	NSCLC	PTK7 may be used as a single biomarker for predicting the efficacy of anti-PD-1 treatment in patients with metastatic NSCLC.	transcriptome analysis	(133)

## 7 The potential of PTK7 in cancer diagnosis and imaging

The diagnostic value of PTK7 is closely linked to its specific expression in cancer cells and its application in the use of aptamers. The two-step strategy of initially selecting cancer cell-specific aptamers and then identifying the target proteins they bind to was discovered over a decade ago. PTK7 has been identified as a potential biomarker for T-ALL (134). Combining PTK7 with other T cell markers has shown promise in detecting minimal residual disease of T-ALL in bone marrow (76). Notably, PTK7-based cancer diagnostics have become increasingly effective in recent years with advancements in molecular diagnostic techniques (135–139). The value of PTK7-targeted aptamers in detecting circulating tumor cells (CTCs) has also been consistently demonstrated (140, 141). While these findings have not yet been translated into clinical applications, the potential significance of PTK7 and PTK7-targeted aptamers in cancer cell detection and diagnosis should not be overlooked.

Another significant area of research focused on PTK7 is cancer imaging. Currently, the quantification of PTK7 with (18)F-Tr-Sgc8 has demonstrated promising clinical translational value, confirming the feasibility of targeting PTK7 for cancer imaging (142). In lymphoma and melanoma, PTK7-targeting aptamer-fluorescent and -radiolabelled probes have shown potential as high-quality molecular imaging agents (143). In T-ALL, a dual-aptamer (Sgc8c and ATP aptamers)-functionalized graphene oxide complex efficiently internalized into target cells, inducing strong fluorescence emission (144). Meanwhile, Sgc8c aptamer-mediated two-photon absorption (TPA) fluorescent organic dots (O-dots) have shown the potential to be an *in vivo* targeting imaging tool with high specificity and efficiency (145). In CRC xenograft models, the Sgc8-c aptamer conjugated with Alexa Fluor 647 fluorophore has also demonstrated clinical translational value (146). Supported by extensive preclinical studies, the first human whole-body dynamic pharmacokinetics study of aptamer targeting the anti-PTK7 sgc8 aptamer was published in 2023, further confirming the feasibility of molecular imaging techniques targeting PTK7 (147). As tumor-derived extracellular vesicles (EVs) have become a new hot spot in cancer research, the potential of PTK7 and PTK7-targeting aptamers for detecting extracellular vesicles has also been explored. For example, by identifying PTK7, a total internal reflection fluorescence assay for PTK7-exosomes could sensitively detect exosomes and distinguish target tumors from controls (148). Furthermore, a recent study has targeted the development of an electrochemical aptasensor for detecting tumor-derived EVs, showing great potential in clinical diagnostics (149).

It is evident that the application of PTK7 in cancer diagnosis and imaging has not yet entered clinical practice, indicating that there is still a long way to go in this area.

## 8 Discussion

Over the past two decades, our understanding of the function of PTK7 has significantly advanced, shedding light on its role in human cancer. From initial expression patterns to molecular mechanisms, regulatory networks, and ongoing clinical trials of drugs, research on PTK7 in cancer has progressed through three major trends. Currently, extensive studies across various human cancer types have provided insight into PTK7 expression changes in different tissues and its preliminary clinical significance. Despite numerous preclinical studies and clinical trials targeting PTK7 in cancer therapy, there has been no significant impact on the clinical treatment paradigm for either cancer type.

However, the future of anti-cancer therapy targeting PTK7 remains promising. While PTK7-based treatment modalities have faced challenges in the past, particularly in the era before cancer immunotherapy became mainstream, advancements in specific recognition, drug delivery, and physical therapy using PTK7-targeted aptamers have the potential to revolutionize cancer treatment. Progress in PTK7-directed ADCs, although showing emerging efficacy, has yet to demonstrate significant survival benefits in patients. The therapeutic potential of other strategies, such as PTK7 monoclonal antibodies, remains uncertain without pan-cancer species trials. Encouragingly, the advent of immunotherapy in recent years has opened new possibilities for PTK7-targeted treatments, as evidenced by its application in CAR-T therapy.

For PTK7-targeted therapies to be a viable option for clinical cancer treatment, it is essential to enhance safety, minimize toxic side effects, and accurately identify the adaptive patient population. Further preclinical studies are needed to investigate PTK7’s involvement in cancer development, addressing the current lack of understanding of molecular interactions and uncovering new signaling pathways with therapeutic relevance. Exploring PTK7’s role in the cancer immune microenvironment is crucial for the development of PTK7-based anticancer immunotherapy. Moreover, investing in cancer molecular diagnostics and imaging will enhance the utilization of PTK7’s unique properties in cancer treatment.
